# Profiles and predictive value of cytokines in children with human metapneumovirus pneumonia

**DOI:** 10.1186/s12985-022-01949-1

**Published:** 2022-12-10

**Authors:** Wen-qing Xiang, Lin Li, Bing-han Wang, Ahmed Faisal Ali, Wei Li

**Affiliations:** 1 Department of Clinical Laboratory, The Children’s Hospital, Zhejiang University School of Medicine, National Clinical Research Center for Child Health, 3333 Binsheng Road, Hangzhou, 310052 People’s Republic of China; 2 School of Public Health, Zhejiang University School of Medicine, Hangzhou, 310052 People’s Republic of China

**Keywords:** Human metapneumovirus, Cytokines, Children, Pneumonia, Predictive value

## Abstract

**Background:**

Human metapneumovirus (HMPV) is an important cause of respiratory tract infections in young children. Early innate immune response to HMPV is focused on induction of antiviral interferons (IFNs) and other pro-inflammatory cytokines that are critical for the formation of adaptive immune responses. To evaluate the predictive value of Th1/Th2 cytokines which include IL-2, IL-4, IL-6, IL-10, INF-γ and TNF-α in pneumonia caused by HMPV.

**Methods:**

A retrospective study was performed among 59 pneumonia pediatric patients with HMPV infection and 33 healthy children as the control cohort, which was detected by the immunofluorescence assay, and the Th1/Th2 cytokines were measured by flow cytometry. 131 children infected with Influenza virus A (IVA) and 41 children infected with influenza virus B (IVB) were detected by RT-PCR assay in throat swabs.

**Results:**

When compared with the healthy children, children who were infected with HMPV pneumonia had a significantly lower level of IL-2 (*p* < 0.001) and higher levels of IL-4 (*p* < 0.001), IL-6 (*p* = 0.001), IL-10 (*p* < 0.001), and IFN-γ (*p* < 0.001). Compared with patients diagnosed with IVA or IVB infection, HMPV-positive patients had significantly higher levels of IL-4 (*p* < 0.001 and < 0.001), IFN-γ (*p* < 0.001 and < 0.001), and TNF-α (*p* < 0.001 and 0.016). Moreover, compared with IVA patients, HMPV-positive patients had a significantly lower level of IL-6 (*p* = 0.033). Finally, when comparing cytokine levels among the patients with HMPV pneumonia, IL-6 and TNF-α levels were found to be significantly higher in the severe group than the mild group (*p* = 0.027 and 0.049). The IL-6 and TNF-α were used to differentiate between mild symptoms and severe symptoms in children diagnosed with HMPV pneumonia with an AUC of 0.678 (95% CI 0.526–0.829) and 0.658 (95% CI 0.506–0.809), respectively.

**Conclusion:**

Our study indicated that difference in cytokine trends depending on the virus species. The levels of IL-4, TNF-α and IFN-γ were significantly distinguished in children infected with HMPV versus IVA and IVB. IL-6 and TNF-α may be helpful in assessing the severity and prognosis of HMPV infection.

**Supplementary Information:**

The online version contains supplementary material available at 10.1186/s12985-022-01949-1.

## Introduction

Human metapneumovirus (HMPV) is a negative-sense, single-stranded RNA virus that belongs to *Metapneumovirus* genus within the family *Pneumovirinae*. It was first isolated from a pediatric patient in the Netherlands in 2001 [1]. It is also one of the most common viruses causing acute upper and lower respiratory tract infections in children, immunocompromised individuals, and elderly [2–5]. HMPV infection most commonly occurs in children below 2 years old [6]. The virus usually causes a series of severe diseases, such as pneumonia and bronchiolitis, and is associated with poor prognosis [7–10]. HMPV is mainly divided into two major groups, A and B, which are further subdivided into four sub-groups, namely A1, A2, B1, and B2 [11, 12]. Recently, more HMPV subtypes were identified (A2c, A2b1, and A2b2) [13].

HMPV infection is one of the major causes of pneumonia in children that is associated with high morbidity and mortality [4, 14]. A rapid and correct diagnosis is crucial for the treatment of HMPV pneumonia. In clinical practice, real-time polymerase chain reaction (RT-PCR) is used to directly detect HMPV DNA in children with pneumonia due to high specificity and sensitivity of the test [15, 16]. However, previous studies have shown that pneumonia caused by HMPV is often combined with other respiratory viruses, such as respiratory syncytial virus (RSV), influenza virus, etc. So the detection of HMPV alone is not sensible to establish the relationship between HMPV and pneumonia in case of co-infection [16, 17]. HMPV preferentially targets ciliated epithelial cells of the human respiratory tract and causes a broad spectrum of respiratory illnesses [18]. The previous study showed that inflammasomes play an important role in hMPV-mediated lung disease and antiviral responses in severely infected children. Level of the pro-inflammatory cytokine IL-18 was significantly upregulated in hMPV-infected children [19]. It is well-known that Th1/Th2 cytokines were crucial in anti-infection immunity after virus infection [20], and our previous study suggested that serum Th1/Th2 cytokines were effective biomarkers to diagnose whether patients afflicted by Gram-negative bacteria [21, 22]. Since serum Th1/Th2 profiles and levels are altered in infected patients, they can be used to quickly identify infectious diseases exist in the early stage and determine the severity of the disease. In this article, we studied the clinical application value of Th1/Th2 cytokines as a reference of auxiliary diagnostic and assessment of disease severity and prognosis of HMPV infection in children with pneumonia.

## Materials and methods

### Study design and patients

We conducted a retrospective study among the children who were diagnosed with pneumonia from May 2012 to June 2019. The study included 59 children with HMPV pneumonia and 33 healthy children as the control cohort. At the same time, 131 children infected with IVA and 41 children infected with IVB were also enrolled in this study. This study has been approved by the medical ethics committee of Children’s Hospital of Zhejiang University School of Medicine. Written informed consents were obtained from parents or guardians of the patients involved in the study. Patients who met the following criteria were enrolled: (1) children under the age of 5 years; (2) primarily diagnosed with pneumonia [23]; (3) children ruled out other respiratory pathogen infections. At the onset of pneumonia, throat swab specimens and blood samples were taken for microbiological analyses and serum Th1/Th2 cytokines determination. According to guidelines for the management of community-acquired pneumonia in children of the People’s Republic of China (2013 Edition) [24], the HMPV-positive patients were divided into 2 groups: mild and severe group.

### Detection of HMPV

HMPV was detected by immunofluorescence assay (Diagnostic Hybrids INC, Ohio, USA) in throat swabs. All operations were conducted according to the manufacturer’s instructions.

### Detection of IVA and IVB

Influenza virus A (IVA) and influenza virus B (IVB) were detected by RT-PCR assay (liferiver, China) in throat swabs. All operations were conducted according to the manufacturer’s instructions.

### Measurement of serum cytokines

1 mL blood sample was collected from every child, and the blood samples were centrifuged at 1000 g for 20 min. The serum was carefully harvested, the Th1/Th2 cytokines were then measured by FACScaliburTM flow cytometer (Becton Dickinson, San Jose, CA, USA). Concentrations of IL-2, IL-4, IL-6, IL-10, tumor necrosis factor α (TNF-α), and interferon γ (IFN-γ) were quantitatively determined by the CBA kit-BDTM CBA Human Th1/Th2 Cytokine Kit II (BD Biosciences, San Jose, CA). The minimal and maximum limits of detection for all six cytokines were 1.0 and 5000 pg/mL, respectively.

### Statistical analysis

The measurement data were selected to test normality using the Shapiro–Wilk test or the Kolmogorov–Smirnov test depending on the sample size. The t-test or the Wilcoxon rank-sum test was used to test for differences in cytokines across influenza populations according to their normality. Non-parametric multiple group component differences were tested using the Kruskal–Wallis test and further adjusted for *p* values for two-by-two comparisons using Holm’s method. The receiver operating characteristic curve (ROC) was used to analyze the results of mild and severe groups, and the sensitivity and specificity were calculated. The area under the receiver operating characteristic curve (AUC) was used to evaluate the diagnostic effect. The closer the AUC is to 1.0, the better the prediction. The AUC between 0.7 and 0.9 shows moderate accuracy of prediction. When the AUC is above 0.9, the accuracy is relatively high. Statistical analyses were completed using R 4.1.2. We reported 2-sided *p* values, and *p* < 0.05 was considered statistically significant.

## Results

From May 2012 to June 2019, a total of 59 patients tested positive for HMPV, about 75% of the patients were below 6 months old, and 92.4% of the patients were below 2 years old.

**Fig. 1:**
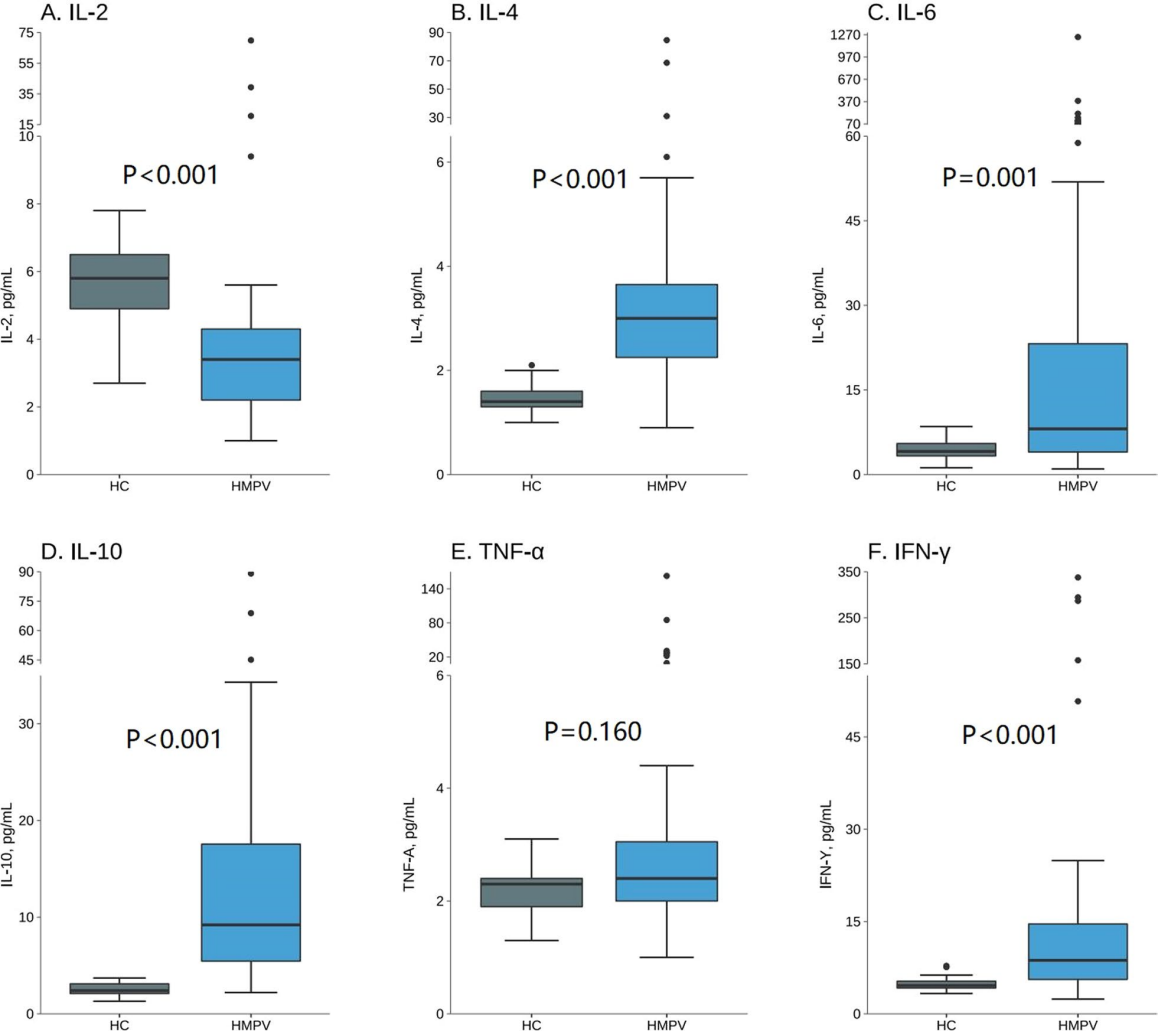
Serum cytokine levels in the control group (N = 33) and HMPV pneumonia group (N = 59). **A**–**F** represent IL-2, IL-4, IL-6, IL-10, TNF-α, IFN-γ levels, respectively. *Control: healthy children group (HC), HMPV Human metapneumovirus

The Th1/Th2 cytokines used in this study are cytokine profile inspection projects in our hospital and the reference ranges for normal values of IL-2, IL-4, IL-6, IL-10, TNF-α, IFN-γ were 1.1-9.8pg/ml, 0.1-3.0pg/ml, 1.7-16.6pg/ml, 2.6-4.9pg/ml, 0.1-5.2pg/ml, 1.6-17.3pg/ml respectively. The levels of six cytokines in the HMPV pneumonia group and normal control group are shown in Fig. 1 and Additional file 1: Table S1. HMPV pneumonia group had lower IL-2 (median levels, pg/ml: 3.40 vs. 5.80, *p* < 0.001), higher IL-4 (median levels, pg/ml: 3.00 vs. 1.40, *p* < 0.001), IL-6 (median levels, pg/ml: 8.10 vs. 4.10, *p* = 0.001), IL-10 (median levels, pg/ml: 9.20 vs. 2.40, *p* < 0.001) and IFN-γ (median levels, pg/ml: 8.70 vs. 4.60, *p* < 0.001), whereas no significant difference of TNF-α levels was found between the two groups (median levels, pg/ml, 2.40 vs. 2.30, *p* = 0.160).

**Fig. 2:**
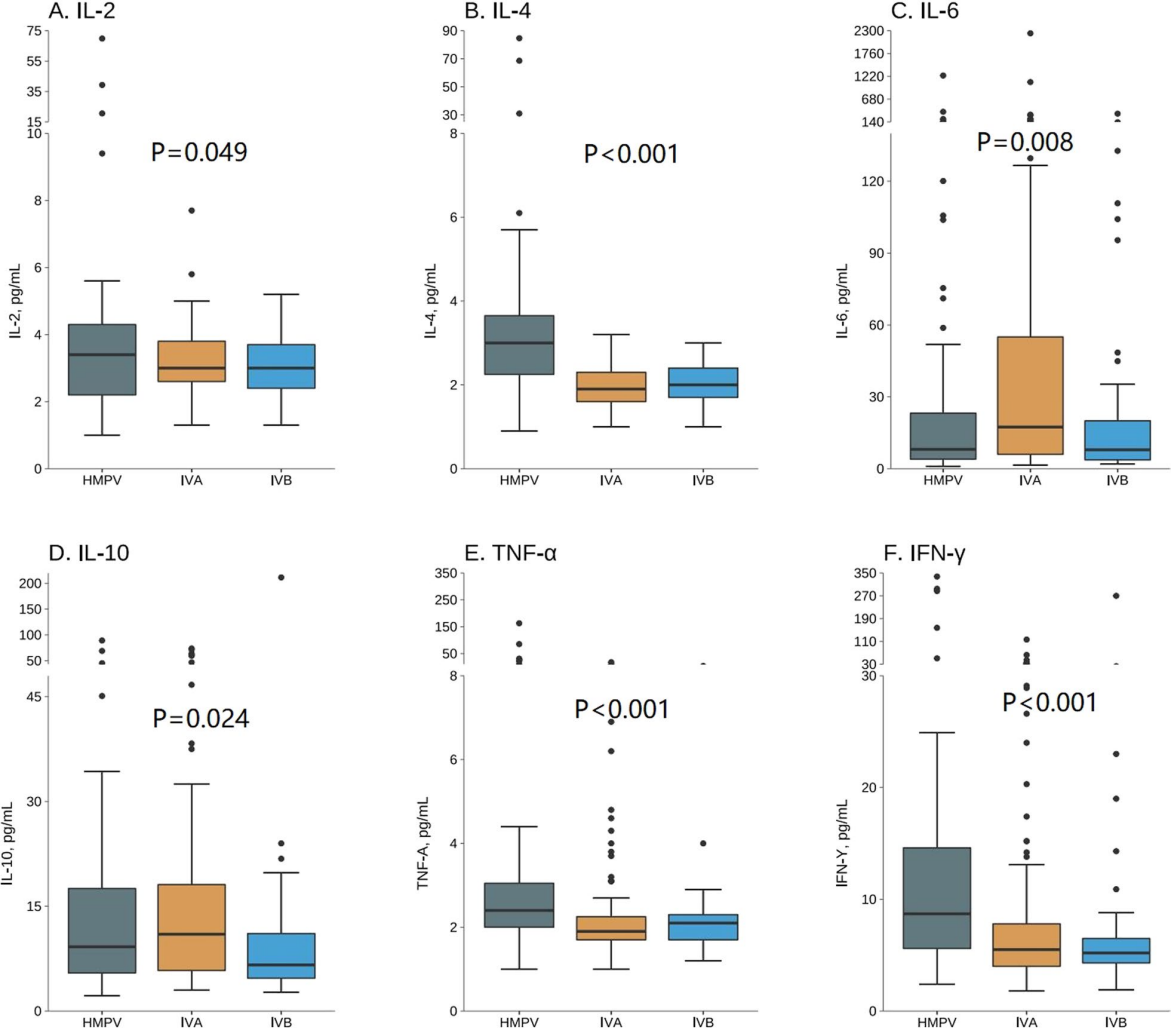
Serum cytokine levels in children with IVA (N = 131), IVB (N = 41), or HMPV (N = 59) infection. A, B, C, D, E and F represent IL-2, IL-4, IL-6, IL-10, TNF-α, IFN-γ levels, respectively. *IVA: Influenza virus A, IVB: Influenza virus B, HMPV: Human metapneumovirus

In order to confirm the differences of cytokines profiles between HMPV and influenza virus, 131 IVA-positive and 41 IVB-positive patients were enrolled in this study, and six cytokines were identified in IVA-positive or IVB-positive children. The cytokine levels in the HMPV, IVA, and IVB groups were shown in Fig. 2 and Additional file 1: Table S1. Compared with IVA and IVB patients, HMPV-positive patients had significantly higher levels of IL-4, TNF-α, and IFN-γ (median levels, pg/ml, IL-4: HMPV = 3.00, IVA = 1.90 (*p* < 0.001), and IVB = 2.00 (*p* < 0.001); TNF-α: HMPV = 2.40, IVA = 1.90 (*p* < 0.001), and IVB = 2.10 (*p* = 0.016); IFN-γ: HMPV = 8.70, IVA = 5.55 (*p* < 0.001), and IVB = 5.20 (*p* < 0.001)). Compared with IVA patients, patients with HMPV had a significantly lower level of IL-6 (median levels, pg/ml, 8.10 vs. 17.40, p = 0.033). In addition, compared with IVA patients, IVB-positive patients had significantly lower levels of IL-6 (median levels, pg/ml, 7.90 vs. 17.40, *p* = 0.033) and IL-10 (median levels, pg/ml, 6.60 vs. 11.00, *p* = 0.017). No difference was found in IL-2 among the three groups (median levels, pg/ml, HMPV = 3.40, IVA = 3.00, IVB = 3.00, *p* = 0.449).

**Fig. 3:**
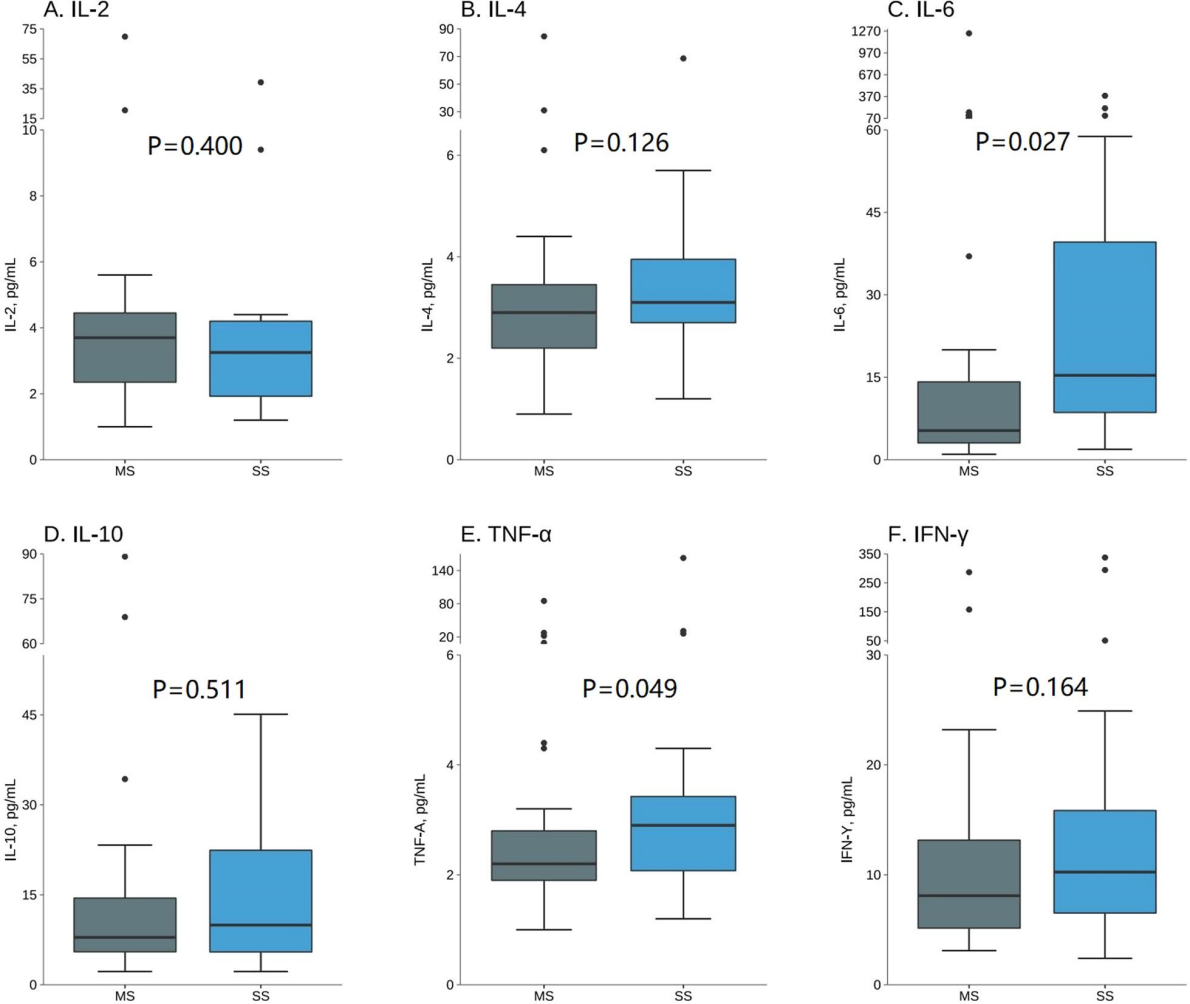
Serum cytokine levels in HMPV-positive children with mild symptom (MS, N = 39) and severe symptom (SS, N = 20). **A**–**F** represent IL-2, IL-4, IL-6, IL-10, TNF-α, IFN-γ levels, respectively

Among the HMPV-positive patients, 39 patients comprised the mild group, and 20 patients comprised the severe group. As shown in Fig. 3 and Additional file 1: Table S1, IL-6 and TNF-α were found to be significantly higher in the severe group than mild group (median levels, pg/mL IL-6:15.35 vs. 5.30, *p* = 0.027; TNF-α: 2.90 vs. 5.30, *p* = 0.049). No significant differences of other inflammatory cytokines (IL-2, IL-4, IL-10, and IFN-γ) levels were found between these two groups (median levels, pg/mL, IL-2: severe = 3.25, mild = 3.70, *p* = 0.400; IL-4: severe = 3.10, mild = 2.90, *p* = 0.126; IL-10: severe = 9.95, mild = 7.90, *p* = 0.511; IFN-γ: severe = 10.25, mild = 8.10, *p* = 0.164).

**Fig. 4:**
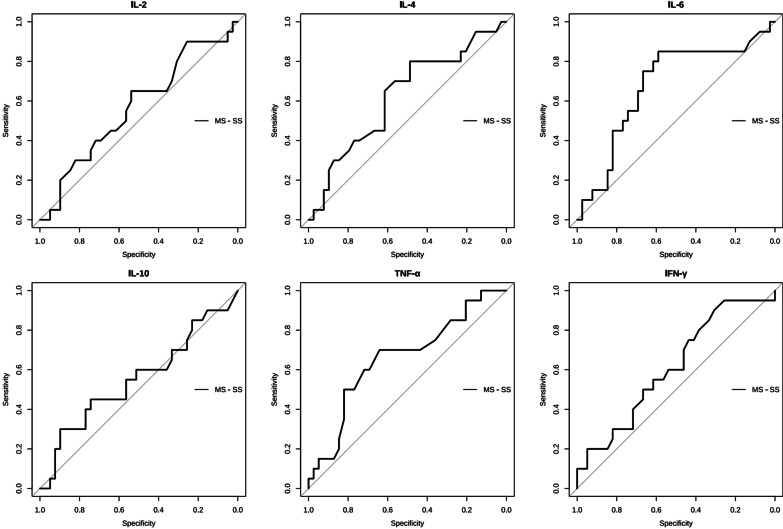
ROC curve of multiple cytokines prediction model for differentiating between mild symptom (MS, N = 39) and severe symptom (SS, N = 20) HMPV pneumonia. **A**–**F** represent the ROC curve of IL-2, IL-4, IL-6, IL-10, TNF-α, IFN-γ, respectively

To confirm the differential diagnostic value of inflammatory cytokine levels in the severity of HMPV pneumonia in children, we used ROC-analysis to compare the six cytokines (Fig. 4). The analysis results indicated IL-6 and TNF-α were effective biomarkers to differentiate between mild and severe symptoms in children diagnosed with HMPV pneumonia with an AUC of 0.678 (95% CI 0.526–0.829) and 0.658 (95% CI 0.506–0.809), respectively. The cut-off points are calculated through the corresponding points of the ROC curve, and the points at which (sensitivity + specificity − 1) take the maximum value are calculated as the cut-off points. Hence, IL-6 ≥ 7.20 pg/mL had a sensitivity of 85.0% and a specificity of 59.0% for severe HMPV pneumonia. TNF-α ≥ 2.50 pg/mL had a sensitivity of 70.0% and a specificity of 64.1% in differentiating between severe and mild HMPV pneumonia.

## Discussion

We grouped 59 children tested HMPV-positive by age and found that 75% were younger than 6 months and 92.4% were younger than 2 years. Therefore, the target of HMPV attack is mainly children under the age of two, which further narrows the scope of previous studies showing that children under the age of five are the main HMPV infections [25]. Children under the age of two are the major population for prevention and control of metapneumovirus pneumonia.

To confirm the immune status of children with HMPV infection and predictive ability of Th1/Th2 cytokines in children with HMPV pneumonia, 59 children with a single infection of HMPV were enrolled in this study. Compared with the HMPV pneumonia group and healthy control group, the former had a significantly lower level of IL-2 and higher levels of IL-4, IL-6, IL-10, and IFN-γ. In addition, IL-6 and TNF-α showed increased expression with severe group. Unlike previous studies, our results showed that the level of IL-2 was significantly lower in the HMPV-infected group, which may because IL-2 is not only very important for maintaining and generating regulatory T cells but also promotes the general proliferation of T lymphocytes, augmenting natural killer cells activity, and inhibiting the formation of granulocyte-macrophage colony. Hence, after infection, the level of IL-2 often decreases [21, 22]. IL-10 can deactivate the macrophage cells. The function and proliferation of T cells and natural killer cells can directly be inhibited by IL-10. Furthermore, the growth and differentiation of B cells, mast cells, granulocytes, dendritic cells, keratinocytes, and endothelial cells can be regulated by IL-10 [26, 27]. Alvarez et al. reported that up-regulation of IL-10 was found in mice with HMPV infection [28]. IL-6 is a major pro-inflammatory mediator that induces acute-phase responses and contributes to host defense against infection and tissue damage. Respiratory syncytial virus (RSV), which is closely related to HMPV, had demonstrated a positive correlation between high IL-6 level and the severity of RSV [29]. In addition, there was a research showed HMPV induced a more severe disease in mice than that of RSV [30]. These results were, to some extent, associated with higher level of IL-6. Consistent with our findings, IL-6 plays an important role in HMPV pathogenicity, which may account for the level of IL-6 was significantly increased in the severe group [31]. The relationship between TNF-α and HMPV has rarely been mentioned in previous studies, but some studies have shown that the expression of TNF-α is increased in RSV-infected patients [32]. In our research, the expression level of TNF-α was significantly increased in the severe group, the mechanism may be similar to RSV, but needs to be further explored. TNF-α may be helpful to distinguish MS and SS group of HMPV. The previous research showed that excessive expression of TNF-α can activate the signal transducer and activator of transcription 3 (STAT3) pathway through NF-κB-mediated IL-6. Unconstrained TNF-α production leads to the excessive activation of inflammatory cytokines, forming a cytokine storm [33]. This may be the reason for the high expression of TNF-α in the SS group than that of MS group. Consistent with previous studies, our results indicated that IL-4 levels were elevated in HMPV-infected group. IL-4 is a potent inflammatory response activator. Previous studies have shown that IL-6 may increase IL-4 and TNF-α production to induce inflammatory responses during the Th2 differentiation process [34]. However, IL-4 secreted by Th2 helper cells may be inhibited when pro-inflammatory cytokine levels are elevated. On the other hand, increased IL-4 level may induce suppression of pro-inflammatory cytokines during the late immune response phase [31, 32]. This may lead to various sensitivities among cytokines that may be helpful to differentiate multiple respiratory viruses. Interestingly, it is speculated that a balanced Th1 type immunity may lead to the clearance of HMPV through activation of IFN-γ secreted by T cells [35].

The hospitalization rate of HMPV infection was similar to that of influenza, using cytokines as biomarkers may be able to differentiate between HMPV and IV. In the early period of influenza-virus infection, NK cells produce IFN-γ. In the subsequent immune response, T cells are the major producer of IFN-γ. Influenza-specific effector CD8^+^ T cells produce a series of cytokines, including IFN-γ and TNF-α through a variety of antigen-dependent pathways [36]. Due to less studies on the differences of cytokine profiles between HMPV and IV, our findings suggested that the levels of IFN-γ and TNF-α are higher than those of influenza virus may be the two viruses have different inflammatory mechanisms, which require further research. Apart from of IL-6, IL-1β, IFN-γ, TNF-α and IL-8, few studies reported increased levels of IL-4 after influenza virus infection [36, 37]. This rule consists with our findings that HMPV-positive patients had a significantly higher level of IL-4. According to a study on influenza virus A, the levels of IL-6 in the site of initial virus infection were increased persistently for 6 days, this may be related the level of IL-6 in HMPV-positive patients was lower than that of IVA-positive patients [37].

Our study also has a little limitation. The sample size is not large enough and data collection time span is too long may affect the consistency of results. In further studies, we plan to increase the detection range and number of samples. In addition, the detection capacity of antigen assay we used in diagnosing HMPV is slightly lower than the gold standard PCR test.

## Conclusion

In conclusion, the majority of children diagnosed with HMPV were below 6 months old. Our study suggested that difference in cytokine trends depending on different virus species. The children with HMPV infection have significantly lower level of IL-2 and higher levels of IL-4, IL-6, IL-10, and IFN-γ when compared with health children. The levels of IL-4, TNF-α and IFN-γ were significantly different in children infected with HMPV versus IVA and IVB. The level of IL-6 was significantly higher in IVA patients than in HMPV patients, which may be able to distinguish HMPV and IVA. IL-6 and TNF-α may be helpful in assessing the severity and prognosis of HMPV infection.

## Supplementary Information

**Additional file 1. Table S1.** Serum cytokine levels in each group (Median, pg/ml).

## Data Availability

All datasets generated in this study are included in the manuscript and/or supplementary files.
