# Supplementary material for: Profiles and predictive value of cytokines in children with human metapneumovirus pneumonia
Source: Virol J. 2022 Dec 10;19:214. doi: 10.1186/s12985-022-01949-1 (PMC9741804; doi:10.1186/s12985-022-01949-1)
Supplement: Supplementary file 1 — Additional file 1. Table S1. Serum cytokine levels in each group (Median, pg/ml). [file 12985_2022_1949_MOESM1_ESM.docx]

**Table. S1** Serum cytokine levels in each group (Median, pg/ml)

|  | **IL-2** | **IL-4** | **IL-6** | **IL-10** | **IFN-γ** | **TNF-α** |
| --- | --- | --- | --- | --- | --- | --- |
| **HC**  **HMPV**  **IVA**  **IVB**  **HMPV-MS**  **HMPV-SS** | 5.8(2.7-7.8)  3.4(1.0-69.8)  3.0(1.3-7.7)  3.0(1.3-5.2)  3.7(1.0-69.8)  3.25(1.2-39.3) | 1.4(1.0-2.1)  3.0(0.9-84.6)  1.9(1.0-3.2)  2.0(1.0-3.0)  2.9(0.9-84.6)  3.1(1.2-68.6) | 4.1(1.2-8.5)  8.1(1.0-1238.4)  17.4(1.5-2236.9)  7.9(2.0-335.8)  5.3(1.0-1238.4)  15.35(1.9-380.4) | 2.4(1.3-3.7)  9.2(2.2-89.1)  11.0(3.0-73.7)  6.6(2.7-211.4)  7.9(2.2-89.1)  9.95(2.2-45.1) | 4.6(3.3-7.8)  8.7(2.4-337.6)  5.5(1.8-116.7)  5.2(1.9-270.0)  8.1(3.1-286.8)  10.25(2.4-337.6) | 2.3(1.3-3.1)  2.4(1.0-162.7)  1.9(1.0-17.8)  2.1(1.2-4.0)  2.2(1.0-85.2)  2.9(1.2-162.7) |

HC: Healthy Children group

HMPV: Human metapneumovirus

IVA: Influenza virus A

IVB: Influenza virus B

HMPV-MS: Human metapneumovirus-Mild System

HMPV-SS: Human metapneumovirus-Severe System
